# Validation of General Anxiety Disorder (GAD-7) questionnaire in Spanish nursing students

**DOI:** 10.7717/peerj.14296

**Published:** 2022-11-01

**Authors:** Sergio Martínez-Vázquez, Juan Miguel Martínez-Galiano, Rocío Adriana Peinado-Molina, Belén Gutiérrez-Sánchez, Antonio Hernández-Martínez

**Affiliations:** 1 Universidad de Jaén, Jaén, Spain; 2 Ciberersp, Madrid, Spain; 3 Universidad de Castilla La Mancha, Ciudad Real, Spain

**Keywords:** Validation, General anxiety disorder, GAD-7 questionnaire, Nursing students

## Abstract

**Background:**

Nursing students are at risk of anxiety during their university education.

**Objective:**

To determine the psychometric characteristics of the General Anxiety Disorder (GAD-7) questionnaire in a population of university nursing students.

**Methods:**

A cross-sectional study was carried out with 170 students at the University of Jaen (Spain) in 2022. An online questionnaire was administered that included sociodemographic and student profile variables, the GAD-7 questionnaire, and the Goldberg anxiety subscale. An exploratory factor analysis (EFA), validation with convergence, and reliability analysis using Cronbach’s *α* were performed.

**Results:**

The EFA identified a single component that explained 63.50% of the variance. It was positively correlated with the Goldberg anxiety subscale (*r* = 0.653; *p* < 0.001). A statistically significant association was observed with academic year, gender, and having experienced an anxiety crisis (*p* < 0.005). Internal consistency with Cronbach’s *α* was 0.903.

**Conclusion:**

The GAD-7 presents appropriate psychometric characteristics for use in a university population of nursing students. It is capable of detecting symptoms and generalized anxiety disorder, making it a useful and simple tool for detecting anxiety-related problems in this population.

## Introduction

Nursing students are at risk of anxiety during their university education (Simpson & Sawatzky, 2020). The combination of theoretical and practical training can contribute to the development of anxiety and its symptoms in students (Collado-Boira et al., 2020; Neal-Boylan, 2010; Rodríguez-Almagro et al., 2021; Romo-Barrientos et al., 2020; Savitsky et al., 2020; Savitsky et al., 2021). Furthermore, anxiety is a common problem among university students, with prevalence ranging from 20.7% to 42.8%, varying with clinical placement, course simulations, and during the pandemic (Hasanpour et al., 2021; Mulyadi et al., 2021; Savitsky et al., 2020). When considering only severe symptoms, the prevalence is 13% (Savitsky et al., 2020), which highlights its public health relevance among the university population.

The problem generated by anxiety in students has a direct impact on academic performance and learning capacity, as well as on general mental health, and may even contribute to the appearance of “burnout” or the risk of suicidal ideation (Alexandrino-Silva et al., 2009; Asfaw et al., 2020; Eisenberg et al., 2007; Gallego-Gómez et al., 2020; Hasanpour et al., 2021; Mulyadi et al., 2021; Ranse, Ranse & Pelkowitz, 2018; Savitsky et al., 2020; Taylor, Eost-Telling & Ellerton, 2018; Zis et al., 2021). It has been argued that anxiety impairs efficient functioning when learning, depending on the balance between inhibition and shifting (Eysenck et al., 2007). Early detection and care are necessary to cushion its effects on students who experience it and to prevent the development of anxiety in those at risk (Hsiung et al., 2019; Huang et al., 2021).

For all these reasons, easy-to-use instruments applicable to the university are needed for early detection and provision of early care regarding anxiety among nursing students (Hsiung et al., 2019). One of these instruments is the GAD-7 (General Anxiety Disorder) (Löwe et al., 2008). The GAD-7 is a self-administered questionnaire that is widely used for its simplicity and has been previously adapted to the Spanish context (Garcia-Campayo et al., 2010) and used among nursing students and other health degrees from other countries (Alonso et al., 2021; Bártolo, Monteiro & Pereira, 2017; Dhira et al., 2021; Eisenberg et al., 2007; Hinz et al., 2017; Lun et al., 2018; Zhang et al., 2021). In addition, health authorities have recommended its use in the Spanish context and in the general population (Conserjería de Salud. Servicio Andaluz de Salud , 2015). This questionnaire is self-administered and was designed for the first time by Löwe et al. in 2008 (Löwe et al., 2008). It consists of seven items, with four response options, and can detect mild to severe symptoms of generalized anxiety disorder (Löwe et al., 2008).

There is no validated instrument available that can detect the level of anxiety early in a population of Spanish nursing students so that early action can be taken to prevent major complications. In Spain more than 91,000 nurses graduate each year (Instituto Nacional de Estadística (INE), 2021). Considering all the reasons mentioned above, the objective of this study was to validate the GAD-7 and verify whether it is a questionnaire capable of detecting generalized anxiety disorder (GAD) among the population of Spanish Nursing students.

## Materials & Methods

### Design

A cross-sectional descriptive study was carried out during March–May 2022 at the University of Jaen (Spain) to validate the General Anxiety Disorder (GAD-7) questionnaire in university nursing students.

### Sample selection

The study population was students enrolled in the Nursing Degree at the University of Jaen. As an inclusion criterion, the students had to be enrolled in the first or second year in 2022 and excluded third and four years students, as higher courses seem to cope better with anxiety (Gupta, Anupama & Ramakrishna, 2021).

The criteria for carrying out a factorial analysis were used to calculate the sample size. These criteria contemplate 10 subjects for each item (De Vet et al., 2005); therefore, a sample of at least 70 participants was needed.

### Information source and variables

A self-administered online questionnaire was used that was made available to the students in the first or second year of the nursing degree. Sociodemographic variables and characteristics of the university profile of university students were collected. This questionnaire also included two scales that assessed anxiety: The Generalized Anxiety Disorder Scale (GAD-7) and the Goldberg anxiety subscale. The former comprises seven items and was designed by Löwe et al. (2008). It has a high internal consistency and has been culturally adapted in the Spanish context and used among nursing students and other health degrees in other countries (Alonso et al., 2021; Bártolo, Monteiro & Pereira, 2017; Dhira et al., 2021; Eisenberg et al., 2007; Hinz et al., 2017; Lun et al., 2018; Zhang et al., 2021).

The GAD questionnaire consists of seven items (“Feeling nervous, anxious, or on edge?”, “Not being able to stop or control worrying?”, “Worrying too much about different things?”, “Trouble relaxing?”, “Being so restless that it is hard to sit still?”, “Becoming easily annoyed or irritable?” and “Feeling afraid as if something awful might happen?”), with four response options each, where each item scores from 0–3, with 21 being the maximum score, and the presence of mild symptoms detected from five points. This tool allows screening for the presence of generalized anxiety disorder (Löwe et al., 2008). The scale designed by Goldberg et al. was used to determine convergent validity (Goldberg et al., 1988). This scale has been adapted to the Spanish cultural context and has been previously used in our cultural context (Montón et al., 1993) and also in other countries among nursing students (Luo et al., 2019). For this purpose, only the first nine items are included, belonging to the Anxiety subscale (Goldberg et al., 1988).

### Statistical analysis

All statistical analyzes have been performed with SPSS version 24.0 (IBM SSPS, Armonk, NY, USA).

The absolute and relative frequencies were used to describe the qualitative variables, and the mean and standard deviation (SD) to describe the quantitative variables of the sociodemographic data and university profile.

An exploratory factor analysis (EFA) was chosen for construct validity to determine the underlying factors through a principal component analysis (PCA), and was used to explore the set of latent variables or common factors that explain the answers to the items of a test. Before performing the EFA, we analyzed the Kaiser-Meyer-Olkin (KMO) tests and the Bartlett sphericity tests to determine if it was appropriate to apply this analysis. For this to be the case, the KMO must be above 0.6 and as close as possible to 1, and the Bartlett sphericity, which consists of the statistical hypothesis test, must present a *p*-value less than 0.05 to reject the hypothesis null of sphericity and ensure that the factorial model is adequate to explain the data.

In the EFA, we used the Varimax rotation, and the kaiser criterion was used to determine the number of factors to retain, one of the most used criteria. Factors with eigenvalues greater than the unit value were retained.

Within the construct validity, we also analyze the convergent validity through the Pearson correlation coefficient between the GAD scale and Goldberg Scale scores and the relationship between the GAD scale scores with the academic year and other sociodemographic and university profile factors. The results were considered statistically significant when *p* ≤ 0.05. We also analyzed the GAD scale’s predictive capacity on high anxiety scores (Scores >or  = 4 points) of the Goldberg scale using the area under the ROC curve. The reliability analysis was performed using the Cronbach test (*α*) to assess internal consistency (IC). The IC indicates to what extent the questionnaire items correlate and how they fit together and measure the same concept. The *α* is one of the most used measures to evaluate the reliability of a scale (Streiner, Norman & Cairney, 2015; Tabachnick & Fidell, 2013). Its values range from 0 to 1. One of the most accepted rules is to consider *α* > 0.9 as excellent, *α* > 0.8 as good, *α* > 0.7 as acceptable, *α* > 0.6 as questionable, *α* > 0.5 as poor, and *α* < 0.5 as unacceptable.

### Ethical considerations

For the collection of information, approval has been obtained from the Research Ethics Committee of the University Jaen, APR.22/3.PRY. All participants had to read the instructions and freely accept an ad-hoc informed consent before beginning to complete the questionnaire. The data was handled in a disaggregated manner, and the anonymity of the participants was maintained at all times.

## Results

### Characteristics of the study subjects

A total of 170 university nursing students participated. The mean age was 22.1 years (SD = 7.03). Of these 170, 82 were enrolled in the first year and 88 in the second year of the degree. The majority (84.7%) were women (144). Most (130, 76.5%) had chosen nursing as their first option for university education (62 in the first year and 68 in the second year). 63.5% (108) had a scholarship at the time of the study. 83.5% (142) had no previous experience in the clinical setting. The most common reason for choosing nursing was vocation (67.6%, 115), followed by the future options that the degree provides (17.1%, 29).

Most first-year students had suffered an anxiety crisis (shortness of breath, palpitations, chest pressure, dizziness, and fear of losing control or dying) on some occasion (74.4%, 61), while slightly fewer second-year students have presented these anxiety crises (59.1%, 52). Further information that characterizes the sample can be found in Table 1.

**Table 1:** Characteristics of the sample included in a validation study.

**Variable**	**Total**	**Academic year**	
	**N (%)**	**1**st **year (%)**	**2**nd **year (%)**
**Gender**			
Men	25 (14.7)	10 (12.2)	15 (17.0)
Women	144 (84.7)	72 (87.8)	72 (81.8)
Non-defined	1 (0.6)	0 (0.0)	1 (1.1)
**Married status**			
Single	147 (86.5)	73 (89.0)	74 (84.1)
Married	8 (4.7)	5 (6.1)	3 (3.4)
Divorced	2 (1.2)	1 (1.2)	1 (1.1)
Other	13 (7.6)	3 (3.7)	10 (11.4)
**Spiritual beliefs (religiosity)**			
No	59 (3.4)	27 (32.9)	32 (36.4)
Yes, non-practicing	78 (45.9)	35 (42.7)	43 (48.9)
Yes, practicing	33 (19.4)	20 (24.4)	13 (14.8)
**Offspring**			
No	162 (95.3)	79 (96.3)	83 (94.3)
Yes	8 (4.7)	3 (3.7)	5 (5.7)
**Working status (currently)**			
No	155 (91.2)	74 (90.2)	81 (92.0)
Yes	15 (8.8)	8 (9.8)	7 (8.0)
**Nursing as first choice**			
No	40 (23.5)	20 (24.4)	20 (22.7)
Yes	130 (76.5)	62 (75.6)	68 (77.3)
**Scholarship**			
No	62 (36.5)	32 (39.0)	30 (34.1)
Yes	108 (63.5)	50 (61.0)	58 (65.9)
**Previous clinical experience (non-clinical placement)**			
No	142 (83.5)	65 (79.3)	77 (87.5)
Yes	28 (16.5)	17 (20.7)	11 (12.5)
**Previous clinical placement experience**			
No	137 (80.6)	58 (70.7)	79 (89.8)
Yes	33 (19.4)	24 (29.3)	9 (10.2)
**Reason to enroll in nursing**			
Professional future	29 (17.1)	10 (12.2)	19 (21.6)
Vocational	115 (67.6)	58 (70.7)	57 (64.8)
Salary/Payment	1 (0.6)	0 (0.0)	1 (1.1)
International options	1 (0.6)	1 (1.2)	0 (0.0)
First choice not available	16 (9.4)	10 (12.2)	6 (6.8)
Other	8 (4.7)	3 (3.7)	5 (5.7)
**Academic level before nursing degree**			
Primary school	2 (1.2)	0 (0.0)	2 (2.3)
Secondary school	7 (4.1)	4 (4.9)	3 (3.4)
High school	154 (90.6)	75 (91.5)	79 (89.8)
Degree	5 (2.9)	3 (3.7)	2 (2.3)
Master’s degree	2 (1.2)	0 (0.0)	2 (2.3)
**Nursing degree access**			
University access test (after high school)	106 (62.4)	49 (59.8)	57 (64.8)
Exam access test	13 (7.6)	6 (7.3)	7 (8.0)
Job training	48 (28.8)	27 (32.9)	22 (25.0)
Other	2 (1.2)	0 (0.0)	2 (2.3)
**Suffered anxiety crisis**			
No	57 (33.5)	21 (25.6)	36 (40.9)
Yes	113 (66.5)	61 (74.4)	52 (59.1)
**Chronic illness**			
No	145 (85.3)	69 (84.1)	76 (86.4)
Yes	25 (14.7)	13 (15.9)	12 (13.6)

### Exploratory factor analysis

**Figure 1:**
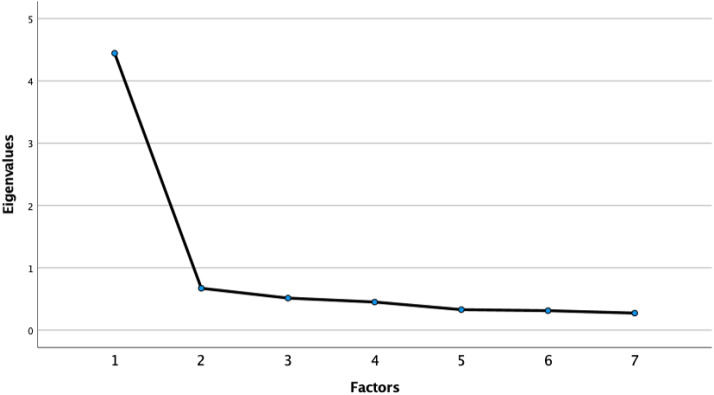
The scree plot of eigenvalues with the Kaiser rule.

The KMO test presented a value of 0.909, and the Bartlett sphericity test was <0.01. Therefore, an EFA was carried out. A single component that contained the 7 items explained 63.50% of the variance. Figure 1 shows the scree plot of eigenvalues with the Kaiser rule. In addition, all anti-image diagonal correlations showed figures higher than 0.884. Table 2 presents the scale items together with their respective factorial weights.

**Table 2:** Response percentage of each item, mean value, and value of the rotated component.

Item	Not at all *n*(%)	Several days *n*(%)	More than half the days *n*(%)	Nearly every day *n*(%)	Mean (SD)	Value Component 1
GAD 1	26 (15.3)	81 (47.6)	36 (21.2)	27 (15.9)	1.38 (0.93)	0.849
GAD 2	79 (46.5)	54 (31.8)	25 (14.7)	12 (7.1)	0.82 (0.93)	0.825
GAD 3	36 (21.2)	60 (35.3)	48 (28.2)	26 (15.3)	1.38 (0.98)	0.806
GAD 4	50 (29.4)	60 (35.3)	40 (23.5)	20 (11.8)	1.18 (0.99)	0.829
GAD 5	82 (48.2)	53 (31.2)	17 (10.0)	18 (10.6)	0.83 (0.99)	0.801
GAD 6	47 (27.6)	73 (42.9)	33 (19.4)	17 (10.0)	1.12 (0.93)	0.735
GAD 7	77 (45.3)	48 (28.2)	29 (17.1)	16 (9.4)	0.91 (1.00)	0.724

Convergent validity and relationship with other sociodemographic and university profile variables.

**Figure 2:**
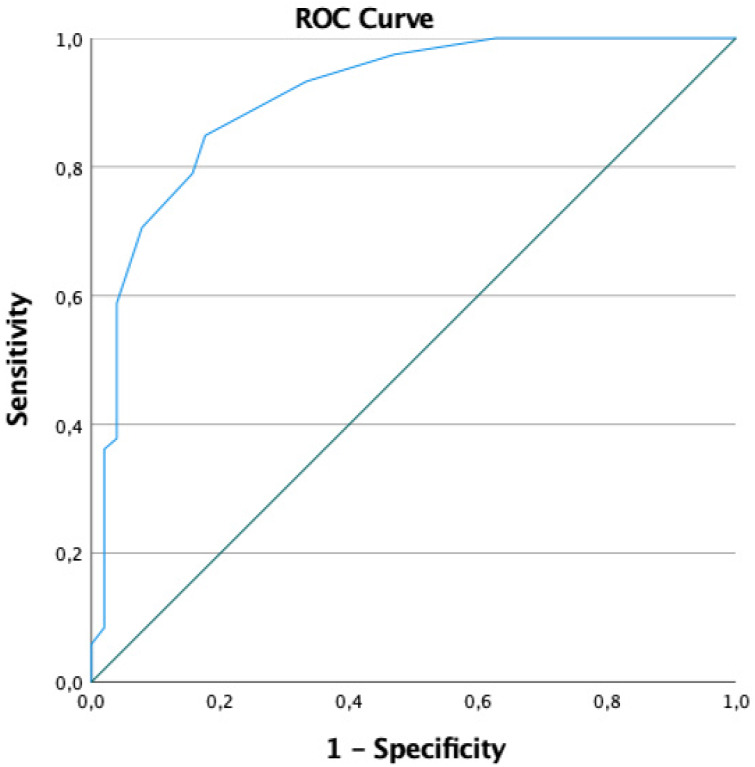
ROC Curve. Predictive capacity of the score in the GAD-7 for anxiety risk using the Goldberg scale.

The Goldberg anxiety subscale was used for the convergent validity analysis, showing a statistically significant correlation with the GAD-7 scale (*r* = 0.653; *p* < 0.001). Next, the relationship between different sociodemographic and university profile variables was studied with the GAD-7 scale, observing a statistically significant association with the academic year, gender, and having experienced an anxiety crisis. First-year students, women, and students with a previous anxiety crisis presented higher scores on the GAD-7 (Table 3). Regarding the predictive capacity of the GAD-7 scale for high scores of the Goldberg scale, an excellent predictive capacity was found with an area under the ROC curve of 0.91 (95% CI [0.86–0.96]) Figure 2.

**Table 3:** Sociodemographic characteristics of the sample and GAD-7 interrelation.

	**Mean (SD)**	***p*** **value**
**Academic year**		
1st year	9.43 (5.54)	**<0.001**
2nd year	5.91 (4.63)	
**Gender**		
Men	5.44 (4.44)	**0.032**
Women	6.56 (4.65)	
**Previous clinical experience (non-clinical placement)**		
No	7.81 (5.54)	0.266
Yes	6.57 (4.37)	
**Subjects passed (nursing degree)**		
Not all	7.44 (5.15)	0.676
All of them	7.78 (5.62)	
**Nursing as first choice**		
No	7.85 (5.93)	0.743
Yes	7.53 (5.21)	
**Scholarship**		
No	8.47 (5.91)	0.113
Yes	7.11 (5.00)	
**Suffered anxiety crisis**		
No	4.95 (4.61)	**<0.001**
Yes	8.94 (5.24)	
**Suffered COVID-19 infection**		
No	7.54 (5.48)	0.876
Yes	7.67 (5.30)	

**Notes.**

Bold: Statistically significant differences

### Internal consistency

For the total scale, the value of *α* was 0.903. All alpha values scored above 0.884 when removing an item. The *α* values for each factor are shown in Table 4.

**Table 4:** Cronbach’s alpha (*α*).

**Variable**	**Cronbach’s alpha (*α*)**
**Total**	0.903
**When removing the item:**	
GAD 1	0.882
GAD 2	0.885
GAD 3	0.888
GAD 4	0.884
GAD 5	0.888
GAD 6	0.896
GAD 7	0.898

## Discussion

The present study was conducted to validate the GAD-7 scale in a population of nursing students and obtained adequate psychometric characteristics. Specifically, internal consistency values above 0.9, considered excellent, and good convergent validity showed that the GAD-7 questionnaire is a reliable and valid tool for assessing anxiety in the Spanish university context.

Of note, the scores obtained were higher for women, first-year students, and for students who had presented a previous anxiety crisis. Regarding gender, similar results have been observed by other authors previously (Gitay et al., 2019; Ren et al., 2021). In the Spanish context, this differentiation by gender is present in other health degrees, such as medicine, where women obtain higher scores on this questionnaire (Santabárbara et al., 2022). Concerning the higher level of anxiety detected among first-year students, it is in line with results from other studies where higher academic years were correlated with a reduction in the scores on this scale (Hwang & Kim, 2022; Melendez Chavarry, 2022). It could be considered, as a future line of research, to implement an intervention program to monitor and control anxiety in these first-year students. Moreover, anxiety levels have been reported as being lower at the time of completing the degree, and incorporation into the professional world further reduces this level of anxiety (Gupta, Anupama & Ramakrishna, 2021). This result could be explained by the improvement in confidence and reduction in anxiety resulting from the acquisition of knowledge and improvement in skills, as shown in other research where students felt less prepared when their training had been of poorer quality (Hernández-Martínez et al., 2021).

The anxiety subscale of the Goldberg Anxiety and Depression Scale (GADS) questionnaire was used to assess the validity of the tool and adequate convergence (Goldberg et al., 1988). The correlation was statistically significant, meaning that higher scores on the GAD-7 scale were associated with higher scores on the Goldberg anxiety subscale. Other authors have used other versions of the GAD-7 to test convergent validity, such as the GAD-2 or the GAD-mini (Byrd-Bredbenner, Eck & Quick, 2020; Byrd-Bredbenner, Eck & Quick, 2021). All these scales have proved their validity; however, more investigations were undertaken with GAD-7, and thus, the GAD-7 scale was selected.

Finally, Cronbach’s *α* was used for internal consistency, obtaining scores above 0.9, which is considered an excellent value. These scores are similar to that obtained by other researchers in the Lithuanian context in health sciences students with an *α* of 0.91 (Pranckeviciene et al., 2022) and a US university population with an *α* of 0.85 (Byrd-Bredbenner, Eck & Quick, 2021).

With the validation of this questionnaire in our study, university professors who teach nursing students have a valid tool for the early detection of generalized anxiety that may require early intervention. This tool is easy to use and versatile and can be applied at different times during university education or even more generalized. It can also be applied in situations that can generate symptoms compatible with anxiety in students, such as exams or clinical rotations (Savitsky et al., 2021; Simpson & Sawatzky, 2020; Stevens, Nussbaum & Blake, 2019; Stinson et al., 2020). It is also a tool recommended by competent authorities in health matters in our country (Conserjería de Salud. Servicio Andaluz de Salud , 2015).

This validated tool in the Spanish educational university context represents a unique opportunity to monitor the mental state of nursing students. The scale can be administered when lecturers perceive students may be experiencing symptoms of anxiety. As lecturers who are trained healthcare professionals, their judgment on symptoms makes them accountable for it. Moreover, other authors have found an association between generalized anxiety and other mental health disorders in the university context and suicidal ideation or severe fatigue, especially in health degrees (Alexandrino-Silva et al., 2009; Asfaw et al., 2020; Eisenberg et al., 2007; Liu et al., 2021), providing extra value to the questionnaire and the validation context.

Regarding possible limitations to consider, although it may be present, we do not believe that recall bias has affected the answers, given that the questions concerning variables of the GAD-7 refer to the previous two weeks. The questionnaire was also anonymous and online, favoring the sincere response of the participants, especially when dealing with sensitive issues such as the expression of symptoms related to mental health, reducing the possible bias associated with it to a minimum (Phillips & Clancy, 1972). As an online questionnaire, there may be a possible limitation in the direct relation to the overuse of mobile devices such as Smartphones. However, the use of technology to collect information facilitates access to the response, and due to the characteristics of the sample, we do not believe there were cases where the skills needed to respond may have influenced the responses obtained (Bernard, 2011). The sample is representative of nursing students in Spain based on age, gender and marital status of the study partipants.

## Conclusions

The validation of the GAD-7 questionnaire in a Spanish population of university nursing students showed adequate psychometric properties, making it an easy-to-use and practical tool for the university nursing educational context.

##  Supplemental Information

10.7717/peerj.14296/supp-1 Supplemental Information 1 Raw data (Spanish)

10.7717/peerj.14296/supp-2 Supplemental Information 2 Raw data (English)

10.7717/peerj.14296/supp-3 Supplemental Information 3 General Anxiety Disorder (GAD-7) Questionnaire

10.7717/peerj.14296/supp-4 Supplemental Information 4 Validation of the GAD-7 questionnaire (Spanish)

10.7717/peerj.14296/supp-5 Supplemental Information 5 Validation of the GAD-7 questionnaire (English)

## Additional Information and Declarations

### Data Availability

The following information was supplied regarding data availability: The raw measurements are available in the Supplementary Files.
