# Supplementary material for: Validation of General Anxiety Disorder (GAD-7) questionnaire in Spanish nursing students
Source: PeerJ. 2022 Nov 1;10:e14296. doi: 10.7717/peerj.14296 (PMC9635356; doi:10.7717/peerj.14296)
Supplement: Supplemental Information 3 [file peerj-10-14296-s003.docx]

Appendix

Appendix A

General Anxiety Disorder (GAD-7) Questionnaire:

| **Please answer honestly according to your situation. Over the last 2 weeks, how often have you been bothered by any of the following problems?** | **Not at all** |  | | |
| --- | --- | --- | --- | --- |
|  |  | **Several days** | **More than half of the days** | **Nearly every day** |
| **Feeling nervous, anxious, or on edge?** |  |  |  |  |
| Option | [] | [] | [] | [] |
| **Not being able to stop or control worrying?** |  |  |  |  |
| Option | [] | [] | [] | [] |
| **Worrying too much about different things?** |  |  |  |  |
| Option | [] | [] | [] | [] |
| **Trouble relaxing?** |  |  |  |  |
| Option | [] | [] | [] | [] |
| **Being so restless that it is hard to sit still?** |  |  |  |  |
| Option | [] | [] | [] | [] |
| **Becoming easily annoyed or irritable?** |  |  |  |  |
| Option | [] | [] | [] | [] |
| **Feeling afraid as if something awful might happen?** |  |  |  |  |
| Option | [] | [] | [] | [] |

Appendix B

Goldberg Anxiety and Depression Scale (Anxiety subscale):

| **Reflect on whether any of the symptoms listed have occurred in the past two weeks. Check only the box that most closely matches each row.** |  |  | | |
| --- | --- | --- | --- | --- |
|  |  | **Yes** | **No** |  |
| **Have you been feeling very excited, nervous, or tense?** |  |  |  |  |
| Option |  | [] | [] |  |
| **Have you been very worried about something?** |  |  |  |  |
| Option |  | [] | [] |  |
| **Have you been feeling very irritable?** |  |  |  |  |
| Option |  | [] | [] |  |
| **Have you had difficulty relaxing?** |  |  |  |  |
| Option |  | [] | [] |  |
| **Have you slept badly, have you had difficulty sleeping?** |  |  |  |  |
| Option |  | [] | [] |  |
| **Have you had headaches or neck pain?** |  |  |  |  |
| Option |  | [] | [] |  |
| **Have you had any of the following symptoms: tremors, tingling, dizziness, sweats, diarrhea?** |  |  |  |  |
| Option |  | [] | [] |  |
| **Have you been worried about your health?** |  |  |  |  |
| Option |  | [] | [] |  |
| **Have you had any difficulty falling asleep, staying asleep?** |  |  |  |  |
| Option |  | [] | [] |  |
