# Supplementary material for: Validation of General Anxiety Disorder (GAD-7) questionnaire in Spanish nursing students
Source: PeerJ. 2022 Nov 1;10:e14296. doi: 10.7717/peerj.14296 (PMC9635356; doi:10.7717/peerj.14296)
Supplement: Supplemental Information 4 [file peerj-10-14296-s004.pdf]

VALIDACIÓN DEL CUESTIONARIO GAD-7 EN ALUMNOS DE ENFERMERÍA Y ESTUDIO DE LOS FACTORES ASOCIADOS A LA ANSIEDAD EN ESTUDIANTES ANTES DE COMENZAR SU PRIMERA ROTACIÓN CLÍNICA

Estimado alumn@, nos dirigimos a ti un grupo de profesionales del campo de la salud, estamos intentando validar una herramienta que detecte el riesgo o incluso la presencia de ansiedad en los estudiantes de enfermería que van a comenzar las prácticas clínicas. Esta herramienta ayudará a detectar casos de desorden de ansiedad generalizada y poder actuar, además, esperamos que tras su validación podamos aplicarla para ver qué factores se asocian con el aumento de la sintomatología o del riesgo de padecer el trastorno y con ello ayudar a la población de estudiantes de enfermería. Si tiene alguna duda puede ponerse en contacto con el investigador principal: Sergio Martínez Vázquez: svazquez@ujaen.es; le agradecemos de antemano su participación en el estudio. Para participar debe aceptar este consentimiento informado marcando la casilla a continuación: (Recuerda que si decides declinar tu participación podrás hacerlo en cualquier momento contactando a los investigadores y que tus datos estarán salvaguardados y anonimizados en todo momento por La Ley Orgánica 3/2018, de 5 de diciembre, de Protección de Datos Personales y garantía de los derechos digitales (LOPD-GDD).

---

*Obligatorio

1. Correo *

____

2. Si está de acuerdo con todo lo expuesto anteriormente, marque la siguiente casilla: *

Selecciona todos los que correspondan.

☐ Sí, estoy de acuerdo

3. Edad *

____

4. Sexo *

Marca solo un óvalo.

- Masculino
- Femenino
- No binario

5. Estado civil *

Marca solo un óvalo.

- Soltero/a
- Casado/a
- Divorciado/a
- Otro

6. ¿Profesas alguna religión? *

Marca solo un óvalo.

- No
- Si, aunque no soy practicante
- Si, soy practicante

7. ¿Tienes hijos/as? *

Marca solo un óvalo.

- Sí
- No

8. Si has respondido afirmativamente a la pregunta anterior especifica cuántos/as *

____

9. ¿Estas trabajando? *

Marca solo un óvalo.

- Sí
- No

10. Nota media del expediente académico (sobre 10) *

____

11. ¿Era Enfermería tu primera opción? *

Marca solo un óvalo.

- Sí
- No

12. ¿Eres beneficiario/a de Beca para estudiar? *

Marca solo un óvalo.

- Sí
- No

13. ¿Tienes experiencia clínica previa? (Sólo se cuenta para este estudio aquella que sea de forma profesional, no otras prácticas clínicas) *

Marca solo un óvalo.

- Sí
- No

14. Si has respondido afirmativamente a la pregunta anterior, especifica en qué ámbito, especialidad o titulación (p.e.: TCAE, Técnico de Rayos, Psicología Clínica, etc.)

15. ¿Has realizado prácticas clínicas anteriormente en algún hospital, centro de salud o centro sanitario? *

Marca solo un óvalo.

- Sí
- No

16. Si has contestado que si has realizado prácticas clínicas ya, por favor, indica para que titulación, curso, etc. la realizaste.

17. ¿Por qué has escogido estudiar enfermería? *

Marca solo un óvalo.

- Salidas profesionales
- Vocación
- Sueldo
- Oportunidades internacionales
- No puede estudiar lo que queria
- Otro: (especificar)

18. He seleccionado "otro" en la pregunta anterior: *

19. Qué nivel de estudios previos máximos tenías antes de acceder a enfermería: (selecciona una) *

Marca solo un óvalo.

- Estudios primarios
- Secundaria
- Bachillerato
- Grado Universitario/Titulación Universitaria
- Máster
- Doctorado

20. ¿Como accediste a los estudios de enfermería? *

Marca solo un óvalo.

- Selectividad
- Prueba de acceso
- Formación profesional
- Otra

21. ¿Has superado todas las asignaturas hasta la fecha de hoy? *

Marca solo un óvalo.

- Sí
- No

22. Si has respondido no a la pregunta anterior dí cuáles *

23. ¿Realizas alguna actividad lúdica que te sirva de relajación? (dibujar, pintar, meditar, cantar, tocar algún instrumento, yoga,etc.,) *

Marca solo un óvalo.

- Sí
- No

24. ¿Padeces alguna enfermedad crónica? *

Marca solo un óvalo.

- Sí
- No

25. Si has respondido sí a la pregunta anterior, especifica cuál: *

____

26. ¿Has tenido alguna vez una crisis de ansiedad? *

Marca solo un óvalo.

- No
- Sí

27. ¿Vives con alguien durante el curso académico? *

Marca solo un óvalo.

- Otros compañer@s de Enfermería
- No, yo vivo solo/a
- Otros compañer@s de otras titulaciones
- Mis compañer@s no son universitarios
- Mixto (compañer@s de titulación y de otras ramas)
- Una familia que no es la mia
- Mi nucleo familiar (padres, hermanos, etc)
- Otro: ____________________

28. ¿Has pasado la infección por coronavirus? *

Marca solo un óvalo.

- Sí
- No

29. ¿Practicas algún deporte habitualmente? *

Marca solo un óvalo.

- Sí
- No

30. ¿Tu domicilio familiar se encuentra en una ciudad o un pueblo? *

Marca solo un óvalo.

- Ciudad
- Pueblo

31. ¿Estás satisfecho/a con tu profesorado de enfermería? *

Marca solo un óvalo.

- Nada satisfecho/a
- Poco satisfecho/a
- Satisfecho/a
- Bastante satisfecho/a
- Muy satisfecho/a

32. ¿Que crees que debe de mejorar en cuanto al profesorado de enfermería se refiere? *

____

____

____

____

____

33. ¿Cumple, en general, con tus expectativas la titulación de Enfermería? *

Marca solo un óvalo.

- Nada
- Poco
- Lo sufiente
- Bastante
- Mucho

34. En general, ¿Estás satisfecho/a con la universidad? (acceso a recursos, horarios, etc.) *

Marca solo un óvalo.

- Nada satisfecho/a
- Poco satisfecho/a
- Satisfecho/a
- Bastante satisfecho/a
- Muy satisfecho/a

35. Si has respondido nada o poco satisfecho/a en la pregunta anterior, especifíca que es lo que no te satisface *

____

36. ¿Que méjorarias de los estudios de enfermeria que estas cursando? (infraestructuras, horarios, material, contenidos, etc.) *

____
____
____
____
____

37. ¿Te sientes motivado/a en tus estudios de enfermería? *

Marca solo un óvalo.

- Sí
- No

38. Por favor responde sinceramente marcando en la tabla la opción que más se ajusta a tu situación. Durante las últimas 2 semanas, ¿qué tan seguido ha tenido molestias debido a los siguientes problemas? Marca únicamente la casilla que más se corresponda en cada fila. *

Selecciona todos los que correspondan.

	Ningún día	Varios días	Más de la mitad de los días	Casi todos los días
**Se ha sentido nervioso(a), ansioso(a) o con los nervios**	☐	☐	☐	☐
**No ha sido capaz de parar o controlar su preocupación**	☐	☐	☐	☐
**Se ha preocupado demasiado por motivos diferentes**	☐	☐	☐	☐
**Ha tenido dificultad para relajarse**	☐	☐	☐	☐
**Se ha sentido tan inquieto(a) que no ha podido quedarse quieto(a)**	☐	☐	☐	☐
**Se ha molestado o irritado fácilmente**	☐	☐	☐	☐
**Ha tenido miedo de que algo terrible fuera a pasar**	☐	☐	☐	☐

39. Reflexione sobre si durante las dos últimas semanas se han presentado algunos de los síntomas que se citan. Marca únicamente la casilla que más se corresponda en cada fila. *

Selecciona todos los que correspondan.

	Sí	No
**¿Se ha sentido muy excitado, nervioso o en tensión?**	☐	☐
**¿Ha estado muy preocupado por algo?**	☐	☐
**¿Se ha sentido muy irritable?**	☐	☐
**¿Ha tenido dificultad para relajarse?**	☐	☐
**¿Ha dormido mal, ha tenido dificultades para dormir?**	☐	☐
**¿Ha tenido dolores de cabeza o nuca?**	☐	☐
**¿Ha tenido alguno de los siguientes síntomas: temblores, hormigueos, mareos, sudores, diarrea?**	☐	☐
**¿Ha estado preocupado por su salud?**	☐	☐
**¿Ha tenido alguna dificultad para conciliar el sueño, para quedarse dormido?**	☐	☐

40. Muchas gracias por su participación, por favor marca esta casilla si te gustaría recibir los resultados del estudio. *

Selecciona todos los que correspondan.

- [ ] Sí, me gustaría recibir los resultados

Este contenido no ha sido creado ni aprobado por Google.

Google Formularios
