# Supplementary material for: Validation of General Anxiety Disorder (GAD-7) questionnaire in Spanish nursing students
Source: PeerJ. 2022 Nov 1;10:e14296. doi: 10.7717/peerj.14296 (PMC9635356; doi:10.7717/peerj.14296)
Supplement: Supplemental Information 5 [file peerj-10-14296-s005.docx]

**VALIDATION OF THE GAD-7 QUESTIONNAIRE IN NURSING STUDENTS AND STUDY OF FACTORS ASSOCIATED WITH ANXIETY IN STUDENTS BEFORE STARTING THEIR PRIMARY CLINICAL ROTATION**

Dear student, a group of professionals in the health field are writing to you. We are trying to validate a tool that detects the risk or even the presence of anxiety in nursing students who are going to start clinical practices. This tool will help detect cases of generalized anxiety disorder and be able to act, furthermore, we hope that after its validation we will be able to apply it to see what factors are associated with increased symptoms or the risk of suffering from the disorder and thereby help the population of nursing students. If you have any questions, you can contact the main researcher: Sergio Martínez Vázquez: [svazquez@ujaen.es](mailto:svazquez@ujaen.es) ; We thank you in advance for your participation in the study. To participate you must accept this informed consent by checking the box below: (Remember that if you decide to decline your participation you can do so at any time by contacting the researchers and that your data will be safeguarded and anonymized at all times by Organic Law 3/2018, of December 5, Protection of Personal Data and guarantee of digital rights (LOPD-GDD).

*Mandatory

1. Mail*

2. If you agree with all of the above, check the following box: *

Select all that apply.

Yes I agree

3. Age *

4. Sex * Mark only one oval.

Male

Female

Non-binary

5. Marital status *

Mark only one oval.

Single

Married

Divorced

Other

6. Do you profess any religion? *

Mark only one oval.

No

Yes, although I am not a practitioner

Yes, I am a practitioner

7. Do you have children? *

Mark only one oval.

Yes

No

8. If you answered yes to the previous question, specify how many *

9. Are you working? *

Mark only one oval.

Yes

No

10. Average mark of the academic record (out of 10) *

11. Was Nursing your first choice? *

Mark only one oval.

Yes

No

12. Are you a beneficiary of a scholarship to study? *

Mark only one oval.

Yes

No

13. Do you have previous clinical experience? (Only the one that is of * is counted for this study - professional way, not other clinical practices)

Mark only one oval.

Yes

No

14. If you answered yes to the previous question, specify in what field, specialty or degree (eg: Nursing Assistant, Ray Technician, Clinical Psychology, etc.)

15. Have you previously carried out clinical practices in a hospital, health center or * health Center?

Mark only one oval.

Yes

No

16. If you have answered that you have already done clinical practices, please indicate for which degree, course, etc. you did it

17. Why have you chosen to study nursing? *

Mark only one oval.

Professional opportunities Vocation

Salary

International opportunities

Unable to study what you wanted

Other: (specify)

18. I have selected "other" in the previous question: *

19. What level of maximum previous studies did you have before entering nursing: *

(select one)

Mark only one oval.

Primary studies Secondary

Baccalaureate

University Degree/University

Master's Degree

Doctorate

20. How did you access nursing studies? *

Mark only one oval.

Selectivity

Entrance test

Professional training

Other

21. Have you passed all the subjects up to today's date? *

Mark only one oval.

Yes

No

22. If you answered no to the previous question, say which ones *

23. Do you do any recreational activity that helps you relax? (draw, paint, meditate, * singing, playing an instrument, yoga, etc.,)

Mark only one oval.

Yes

No

24. Do you suffer from any chronic disease? *

Mark only one oval.

Yes

No

25. If you answered yes to the previous question, specify which one: *

26. Have you ever had an anxiety crisis? *

Mark only one oval.

Yes

No

27. Do you live with someone during the academic year? *

Mark only one oval.

Other Nursing colleagues

No, I live alone

Other colleagues from other degrees

My colleagues are not university students

Mixed (colleagues of degree and other branches)

A ​​family that is not mine

My family nucleus (parents, siblings, etc.)

Other:

28. Have you had the coronavirus infection? *

Mark only one oval.

Yes

No

29. Do you practice any sport regularly? *

Mark only one oval.

Yes

No

30. Is your family home in a city or town? *

Mark only one oval.

City

Town

31. Are you satisfied with your nursing faculty? *

Mark only one oval.

Not at all satisfied

A little satisfied

Satisfied

Quite satisfied

Very satisfied

32. What do you think should be improved in terms of nursing faculty? *

33. In general, does the Nursing degree meet your expectations? *

Mark only one oval.

Nothing

Little

Enough

Quite

A lot

34. In general, are you satisfied with the university? (access to resources, schedules, etc.) *

Mark only one oval.

Not at all satisfied

A little satisfied

Satisfied

Quite satisfied

Very satisfied

35. If you answered not at all or not very satisfied in the previous question, specify what * that does not satisfy you

36. What would you improve from the nursing studies you are studying? (infrastructures, * schedules, material, content, etc.)

37. Do you feel motivated in your nursing studies? *

Mark only one oval.

Yes

No

38. Please answer honestly by marking the option that best suits your *

situation. During the last 2 weeks, how often have you been bothered by the following problems? Check only the box that most closely matches each row.

| Over the last two weeks, how often have you been bothered by the following problems? | Not at all | Several days | More than half the days | Nearly every day |
| --- | --- | --- | --- | --- |
| 1. Feeling nervous, anxious, or on edge | 0 | 1 | 2 | 3 |
| 2. Not being able to stop or control worrying | 0 | 1 | 2 | 3 |
| 3. Worrying too much about different things | 0 | 1 | 2 | 3 |
| 4. Trouble relaxing | 0 | 1 | 2 | 3 |
| 5. Being so restless that it is hard to sit still | 0 | 1 | 2 | 3 |
| 6. Becoming easily annoyed or irritable | 0 | 1 | 2 | 3 |
| 7. Feeling afraid, as if something awful might happen | 0 | 1 | 2 | 3 |

39. Select all that apply. Reflect on whether any of the symptoms listed have occurred in the past two weeks. Check only the box that most closely matches each row.

Next are some specific questions about your health and how you have been eeling in the PAST MONTH.

Have you felt keyed up or on edge?

Yes

No

Have you been worrying a lot?

Yes

No

Have you been irritable?

Yes

No

Have you had difficulty relaxing?

Yes

No

Have you been sleeping poorly?

Yes

No

Have you had headaches or neckaches?

Yes

No

Have you had any of the following: trembling, tingling, dizzy spells, sweating, diarrhoea or needing to pass urine more often than usual?

Yes

No

Have you been worried about your health?

Yes

No

Have you had difficulty falling asleep?

Yes

No

40.

Select all that apply.

Yes, I would like to receive the results

Thank you very much for your participation, please check this box if you would like to receive the results of the study.

This content has not been created or approved by Google.
